# Treatment of Deciduous Molars

**Published:** 1903-12

**Authors:** G. E. Adams

**Affiliations:** South Orange, N. J.


### TREATMENT OF DECIDUOUS MOLARS.[1]

BY DR. G. E. ADAMS, SOUTH ORANGE, N. J.

Mr. President and Gentlemen of the Institute of Stomatology,—The question of treatment of the deciduous teeth is one of vital importance, as these teeth should be held *in situ* until forced from their places by the newly erupting ones.

In the early days of dentistry the deciduous teeth were thought to be of so little importance that they were allowed to decay and were extracted before they had fulfilled one-half of their function as regards time limit.

In a "Guide to Sound Teeth," published in New York in 1838 (Shearjashub Spooner, M.D., author), may be found the following:

"It often happens that the temporary teeth, especially the molars, decay at an early age, and expose the child to severe toothache. Under such circumstances, it is common to extract the painful teeth. We think the practice highly improper, as irregularities are often caused thereby; and that the temporary molar teeth should never be extracted for toothache unless it proceed from inflammation, ulceration, and the formation of gum-boils, in which case they should be removed for fear of mischief to the permanent teeth, which are being formed underneath them, as well as rid the child of pain incurable by other means. If the posterior temporary molar teeth be extracted, the first four permanent molars, which the child usually gets at six or seven years of age, will be very apt to come forward, so as partially to occupy their places. The consequence will be permanent irregularity, for there will not be room anterior to the permanent molars for the permanent teeth springing from the temporary incisors, cuspidati, and bicuspids.

"As the incisors and bicuspids come in before the cuspidati, the irregularity will be very apt to happen to the latter teeth, to remedy which will require the sacrifice of the four posterior permanent bicuspids. We universally destroy the nerves of the temporary molar teeth, for the cure of the tooth in preference to extraction, and recommend the nurse to keep them stopped with gum mastic, or stop them ourselves with cement. The mastic

[1] Read before The New York Institute of Stomatology, June 2, 1903.

answers a very good purpose, as it is insoluble in water. The nerves of the teeth may be destroyed, as a general rule without pain and with least danger, by means of a very little arsenic, as will be seen under the head of 'Stopping and plugging the teeth.' Besides the prevention of the consequences before mentioned by the practice we recommend, the child often keeps these teeth highly useful for mastication."

Remember, gentlemen, this was written sixty-four years ago. In the treatise on extraction, by Professor Thomas C. Stellwagen, published in the "American System of Dentistry," we find the following:

"The deciduous tooth rarely needs to be removed until the permanent successor comes so near the surface as to be readily felt upon piercing the gum with a sharp needle. Teeth of the first dentition, even after severe injuries, will generally recover and serve their purpose until near the time of the appearance of their successors. Portions of the roots of the deciduous teeth denuded, as the result of wasting by absorption or by ulceration and sloughing of the overlying tissues, have frequently been cut off and their remaining ends polished to prevent their causing diseases of the tongue or of the soft tissues of the cheeks in contact with them."

So much for quotations. How frequently are our first services for the child called for when one or more of these deciduous teeth are giving trouble! We find on examination that the approximal surfaces are badly decayed, and possibly the pulps are putrescent, the youngster cannot eat without discomfort because of the pressure against the inflamed gum, and we hardly know how to decide for the best interests of the small patient. Shall we extract the teeth or attempt to conserve their usefulness for a while longer? The conscientious practitioner is sometimes halting between the necessity for retention and the fear of further trouble if they be retained, for the strain of the work, even though it be painless, is quite a factor to be taken into consideration. A little patient is brought to us, thin, pale and nervous, and we are told that it cannot eat, or will not because of the pain experienced, and after a day or two of trying to masticate, the child takes to prepared foods, as one child who ate nothing for several months but cereals and bread soaked in milk, but who after the insertion of a chloropercha dressing was able to eat what her brother and sister did. Examination reveals a growth, highly inflamed, extending to the occlusal surfaces, and the slightest touch produces pain and hemorrhage, and we do not wonder that the child shrinks at the anticipated discomfort.

The initial step in such cases in approximal cavities in the first and second temporary molars extending to the gum, or when the inflamed gum has encroached upon the cavity, is the strangulation of the growth and making it possible to masticate with comfort. This can easily be done by first cauterizing the growth with carbolic acid and forcing a pledget of cotton saturated with chloro-percha into the cavity, for as the chloroform soon evaporates, a firm filling remains which will permit mastication for several weeks if necessary, although the longer it is left after two or three days the wider the separation and the more doubtful the future usefulness of the teeth.

The taste of chloroform in such small quantities is rather pleasant to children, and they frequently ask if they may have "some of that pink stuff." After the lapse of three or four days, the dressing being removed, the cavities may be treated as indicated.

The method of the writer in deciduous teeth with putrescent pulps is, first, the removal of all débris that is present as far as possible without cutting through the enamel, and inserting a small pledget of cotton saturated with Dr. Black's 1, 2, 3, composed of carbolic acid, oil of cinnamon, and oil of wintergreen, and filling the cavity with oxyphosphate or pink gutta-percha if the cavity be not too large. This gutta-percha may be added to from time to time, as it wears down, and the comfort of the little patient is maintained and mastication is assured.

Care is essential that the roots of these teeth be removed at the proper time, so as to guard against the eruption of the permanent teeth out of line.

It is incumbent upon the men of our profession to present to parents the necessity of frequent visits for examination, that the ravages of caries in the deciduous teeth may be retarded. These frequent visits eliminate the possibility of painful and protracted operations, and children grow to look upon the time spent at the dentist's with feelings of pleasure, and take the chair with cheerful and smiling faces; and the bugbear of the dentist is unknown.

